# Knowledge of hypertensive disorders in pregnancy of Moroccan women in Morocco and in the Netherlands: a qualitative interview study

**DOI:** 10.1186/s12884-018-1980-1

**Published:** 2018-08-22

**Authors:** Fatima Ouasmani, Bernice Engeltjes, Bouchra Haddou Rahou, Ouafae Belayachi, Corine Verhoeven

**Affiliations:** 1 Laboratory of Genetic, Neuroendocrinology and Biotechnology, Faculty of Sciences, Ibn Tofail University, Kenitra, Morocco; 2 High Institute of Nursing Professions and Technical Health, Rabat, Morocco; 3 Rotterdam University of Applied Science, Rotterdam, the Netherlands; 4 School of Public Health, University of Montreal, Montreal, Canada; 5 Amsterdam UMC, Vrije Universiteit Amsterdam, Department of Midwifery Science, AVAG, Amsterdam Public Health research institute, Amsterdam, the Netherlands; 6 Department of Obstetrics and Gynecology, Maxima Medical Centre, Veldhoven, The Netherlands

**Keywords:** Hypertensive disorders in pregnancy, Symptoms, Knowledge, Morocco

## Abstract

**Background:**

Hypertensive disorders in pregnancy (HDP) are the most common medical disorders in pregnancy and the greatest single cause of maternal mortality worldwide. Ethnicity appears to be a significant risk factor for pregnancy related mortality and for severe maternal morbidity. Most of the complications caused by HDP may be reduced by early detection and proper management. Health education during antenatal care attendance may play an important role in preventing the disease to aggravate. The purpose of this study was to investigate the status of knowledge that Moroccan pregnant women, both in Morocco and in the Netherlands, have of HDP in terms of symptoms, complications, treatment and management.

**Methods:**

A qualitative research design was used to explore and describe the knowledge of HDP of pregnant Moroccan women. Interviews were held on the basis of a topic list. The interviews were recorded, transcribed, coded and analysed.

**Results:**

Nineteen Moroccan women were interviewed, nine in the Netherlands and ten in Morocco. Half of them never heard about hypertension in pregnancy and had no knowledge of symptoms or alarm signals related to HDP. All women acknowledged the importance of knowledge of HDP because of the possibly dangerous complications. The interviewees stated that information on symptoms, alarm signs and complications is the most important information. Nearly all women stated that communicating information via movies was the most appropriate tool to inform Moroccan pregnant women about HDP.

**Conclusions:**

The knowledge of Moroccan women, living in Morocco or in the Netherlands, of symptoms and alarm signs related to hypertensive disorders of pregnancy was very limited, if not absent. Since early detection provides the opportunity for follow-up management and/or treatment, this may reduce complications of HDP. Therefore, it is important to inform pregnant women about the signs and symptoms of HDP.

**Electronic supplementary material:**

The online version of this article (10.1186/s12884-018-1980-1) contains supplementary material, which is available to authorized users.

## Background

### Preface

This study was carried out as part of a Twinning project between Morocco and The Netherlands. The International Confederation of Midwives (ICM) introduced Twinning as an approach to strengthen midwives associations in 2009. According to the Millennium Development Goals Report (2014) maternal mortality remains unacceptably high across much of the developing world. In attempting to address the maternal mortality rate ICM, with financial support from the Netherlands Ministry of Foreign Affairs (MoFA) developed the project Midwives: Enhancing the Reproductive Health of Women. This project aims to enable midwives to effectively contribute to enhancing the reproductive health of women and reduce maternal and newborn mortality in their countries.

The Dutch Organization of Midwives (KNOV) is committed to reducing mother and newborn deaths through cooperation with sister organizations in countries where the childbirth-related death rate remains high. KNOV and Moroccan sister organization the Association Marocaine des Sages Femmes (AMSF) will exchange knowledge and support each other in the project which aims to improve care for pregnant mothers in Morocco and for pregnant Dutch-Moroccan mothers in the Netherlands.

### Introduction

Hypertensive disorders in pregnancy (HDP) are the greatest single cause of maternal mortality worldwide [1]. It is the most common medical disorder in pregnancy and 7 to 10% of all pregnancies are complicated by hypertension and 2 to 8% by preeclampsia [2, 3]. HDP are an important cause of feto-maternal morbidity and mortality, particularly in developing countries [4]. Most of the complications caused by HDP may be reduced by early detection and proper management [5, 6].

In Morocco, HDP is the second most common direct obstetric cause of maternal mortality, accounting for 18.4% from all deaths and 24.2% compared to the direct causes of death. The main cause of HDP-related maternal mortality is eclampsia with 72.9% followed by preeclampsia (16.7%) [7, 8].

In the Netherlands, eclampsia/preeclampsia is the leading cause of maternal mortality. The risk of gestational hypertension and preeclampsia has been demonstrated to differ by ethnic background [9, 10]. In a nationwide cohort study of severe maternal morbidity in the Netherlands 222 cases of eclampsia were reported, an incidence of 6.2 per 10,000 deliveries. Ethnicity appeared to be a significant risk factor for pregnancy related death and for severe maternal morbidity [11]. The risk of maternal mortality has been reported three times elevated for immigrants as compared to native women in the Netherlands [12, 13]. Ethnic minority groups of non-European origin form 11% of the total population of the Netherlands [14] and migrants from Turkey and Morocco are the two largest Islamic minorities [15]. Recent evidence suggests that a part of complications related to HDP is the result of inadequate knowledge; and negative attitude towards and lack of preventive practice [16]. In the Saving Mothers report HDP constitutes 14% of maternal mortality in developing countries because in its early stage a woman may totally be unaware of its presence [17].

Prenatal care in Morocco is organized at the basic health care facilities level, delivery homes, maternity and mobile contact points; and all pregnant women have free access to antenatal care. Care is provided by midwives, general practitioners and/or gynecologists. Women with normal pregnancies benefit from four prenatal consultations. The proportion of women who received antenatal care was around 77.1% in 2011 compared to 67.8% in 2004. This proportion differs significantly by 91.1% in urban against 62.7% in rural areas [18].

In the Netherlands prenatal care is provided in primary care by midwives in case of uncomplicated pregnancies, or in secondary hospital-based care by obstetricians in case of a complicated pregnancy, If complications during pregnancy or birth occur, such as HDP, midwives will refer a pregnant woman to secondary obstetric-led care. Prenatal care is accessible for every pregnant women and consists of 12 to 14 prenatal consultations. The proportion of women who received adequate prenatal care is around 90%. This proportion differs significantly by western and non-western population living in the Netherlands [19].

Screening for hypertension is simple and inexpensive. Effective methods for prevention of HDP are limited, but health education during antenatal care attendance may play an important role in preventing the disease to aggravate.

Hence, the purpose of this study was to investigate the status of knowledge that Moroccan pregnant women have of HDP, both in Morocco and in the Netherlands, in terms of symptoms, complications, treatment and management.

## Methods

A qualitative research design was used to explore and describe the knowledge of HDP of pregnant Moroccan women living in Morocco or in the Netherlands. In Morocco, women attending antenatal care in health centers at the Rabat region in urban and rural areas were included. In the Netherlands women were selected in five midwifery practices in the city Amsterdam, Rotterdam, Eindhoven and two villages.

Purposive sampling was used both in Morocco and in the Netherlands until saturation was reached. Women who agreed to participate in the study were included in any trimester of pregnancy.

Permission to access the health centers and to conduct the study was obtained from the Ministry of Health in Morocco. In the Netherlands no ethical approval is required regarding this type of research (Central Committee on Research Involving Human Subjects (CCMO) http://www.ccmo.nl, stating ‘Research regarding interviewing people for the collection of data does, in principle, not need to be reviewed by the ethical committee.’). Verbal consent of the participants was sufficient according to the CCMO. All participants were provided with sufficient and understandable information about participation in the study and gave their verbal informed consent to participate before the start of the interview; and this was recorded. Confidentiality and anonymity were ensured.

Semi-structured interviews were conducted using dialectal Arabic in Morocco and the Dutch language in the Netherlands. The interviews lasted no more than 45 min and the following themes were used: knowledge of HDP: knowledge of the disease and knowledge of symptoms of HDP; risk factors for developing HDP, complications and alarm signs; behavior in case of HDP: practice, treatment and the influence of traditional practice; and finally the source of knowledge: source and content of information (See Additional file 1).

The interviews were conducted at the respondents’ residences in Netherlands and in health centers in Morocco and were tape-recorded. After each interview, reflective summaries and clarifications with the participants were undertaken by the interviewer. Verbatim transcription and translation of the interviews were performed in Morocco and in the Netherlands. Transcripts were translated into English before analysis and data were analysed qualitatively using the open coding method as described by Creswell [20]. Successive readings were performed to highlight the key units of meaning that emerged. Then, categories, covering all of these meaning units were defined thus highlighting the most frequently mentioned in connection with the knowledge of HDP. Member checks were performed with the participants to verify the accuracy of the data.

## Results

Between March and July 2016, nineteen Moroccan women were interviewed, nine in the Netherlands and ten in Morocco.

Women were between 20 to 44 years old and their pregnancy age varied between 12 and 37 weeks at the moment of the interview (Table 1). Five participants were primigravida. In Morocco seven out of the ten participants were housewives and came from rural and mountainous areas. In the Netherlands, all women had a paid job and most of the women lived in a city. In Morocco three out of the ten women were illiterate and almost half of them travelled to health centers on foot. The mean time for remoteness to health center was 24 min. In the Netherlands most women had followed higher education and all of the women travelled to the health centers by car. Four women in Morocco were living with their in-laws. In the Netherlands none of the women lived with their in-laws (Table 1). Six of the nine Dutch Moroccan women were born in the Netherlands. One woman migrated with her family from Morocco to the Netherlands when she was 4 years old, the remaining two women were 9 years old when they started living in the Netherlands.

**Table 1:** Socio-demographic and clinical characteristics of study participants

Moroccan Participants	Age (years)	Region	Educational level	Occupation	Persons living in the same household	Gestational age (weeks)	Gestation	Parity	Transportation used to health center	Caregiver	Remoteness to health center (minutes)
M	35–40	Urban	Illiterate	Yes	With husband and children	12	3	2	Public transport	Midwife	5
M	40–45	Rural	Illiterate	No	With husband, children and family in law	28	6	5	Mule	Nurse	30
M	35–40	Rural	Secondary level	No	With husband and children	28	3	2	Public transport	Midwife	25
M	25–30	Rural	Illiterate	No	With husband, children and family in law	13	3	2	Walk (by foot)	Midwife	45
M	25–30	Urban	University level	Yes	With husband	28	1	0	Car	Midwife	5
M	25–30	Rural	Secondary level	No	With husband and child	37	2	1	Car	Midwife	10
M	40–45	Urban	University level	Yes	With husband and children	34	3	2	Friend’s car	Midwife	30
M	20–25	Urban	University level	No	With husband	25	1	0	Walk (by foot)	Midwife	10
M	25–30	Urban	Primary level	No	With husband and family in law	20	1	0	Walk (by foot)	Midwife	5
M	25–30	Urban	Primary level	No	With husband, children and family in law	20	3	2	Walk (by foot)	Midwife	5
Dutch Participants	Age (years)	Since when in the Netherlands	Educational level	Occupation	Persons living in the same household	Gestational age (weeks)	Gestation	Parity	Transportation used to access to health center	Caregiver	Remoteness to health center (minutes)
D	25–30	Born	Secondary	Yes	With husband	22	1	0	Car	Midwife	5
D	35–40	Since 9th year of life	High	Yes	With husband and children	35	4	3	Car	Midwife	10
D	20–25	Born	Secondary	Yes	With husband and child	28	3	1	Car	Midwife	5
D	25–30	Since 4th year of life	High	Yes	With husband	27	1	0	Car	Midwife	11
D	30–35	Born	High	Yes	With husband and child	22	2	1	Car	Midwife	7
D	30–35	Born	High	Yes	With husband and children	18	3	2	Car	Midwife	10
D	25–30	Since 9th year of life	High	Yes	With husband and child	17	2	1	Car	Midwife	10
D	35–40	Born	High	Yes	With husband and children	17	4	3	Car	Midwife	10
D	20–25	Born	Studying High School	Yes	With husband and child	33	2	1	Car	Midwife	20

In the analysis of the transcribed interviews, the following themes and sub-themes, reflecting knowledge of HDP, emerged. The themes are summarized in Table 2.

**Table 2:** Themes and sub-themes

Theme	Sub-themes
1. Knowledge about HDP	1. Knowledge of HDP as a disease 2. Knowledge of symptoms of HDP
	3. Knowledge of alarm signs and complications of HDP 4. Knowledge of treatment for HDP and traditional practices
2. Behavior in case of HDP	1. Practices in case of HDP
3. Information about HDP	1. Source of information 2. Content of information
4. Suggestions	1. Important information to know 2. Most appropriate tool for the communication of information

### Theme 1: Knowledge of HDP

#### Sub-theme: Knowledge of HDP as a disease

When women were asked about the knowledge of high blood pressure in pregnancy, the responses varied. Half of the nineteen women stated they had never heard about hypertension in pregnancy.

“*I have no idea about this disease*” *(M)*

*“No idea, I don’t know really much of this… ”(D)*

*“little actually, because if you do not have it yourself, you’re not going to read about” (D)*

*“I have a low blood pressure myself, I do not know” (D)*

Some women knew high blood pressure is a disease but only some of them distinguished HDP as a disease related to pregnancy. This was due to previous experience of themselves or of a family member. A few women knew the association between high blood pressure and preeclampsia.

*“Yes, I know this disease, it is high blood pressure in pregnancy” (M).*

*“Yes, my husband has this disease, his blood pressure is always high” (M)*

*“I can imagine that high blood pressure can give pre-eclampsia and other complaints to the mother” (D)*

#### Sub-theme: Knowledge of symptoms of HDP

There was a lack of knowledge of HDP symptoms among the majority of the participating women, both in Morocco and in the Netherlands. Half of the women did not know any symptoms at all, and guessed some symptoms.

“*I do not know the symptoms”*. *“No idea” (M)*

*“Maybe .. I think palpitations, my mom also has this sometimes now” (D)*

*“When I think of high blood pressure, I think of stress. That’s the only link I can get now. Not really pregnancy related…*” *(D)*

*“I think they [pregnant women] become swollen?” (D)*

Symptoms cited were headache, oedema, abdominal pain, dizziness and tiredness. No woman was able to list a minimum of three symptoms. One woman was clearly confident that sweating was the symptom of HDP.

*“Yes women have headache and oedema” (M).*

*“Tiredness and headache” (M)*

*“The only complaint is headache” (M)*

*“Many oedema, she swelled completely” (D)*

*“Yes, oedema, shortness of breath, headache?” (D)*

*“Yes, headache and abdominal pain and dizziness”(D)*

#### Sub-theme: Knowledge of alarm signs and complications of HDP

In Morocco, the majority of the women was unable to identify the signs of alarm and danger.

*“I don’t know”. (M)*

“*I heard on the radio that the intense and continuous headaches are among the danger signs».” (M)*

*“My friend had this disease. She was in the emergency room, she had severe headache with vomiting” (M)*

In the Netherlands, most Moroccan women were doubtful about “whether HDP is dangerous to health”.

*“I would not know. No idea if it is a problem for me or the baby…*” *(D)*

*“I think more for the child… not for me. For the baby. All you can get during pregnancy, is all hormonal and it is all different in women. So, I think what matters most, is the baby, yes. Right?” (D)*

*“High blood pressure is not good, also not for the baby” (D)*

*“Yes, the blood supply, I think that’s not working well. If the blood supply is not working, I mean it is the motor of your body. The organs and your baby get no oxygen. …. Yes that’s asphyxia actually…. I have heard it’s dangerous for the baby. The woman has to go to the hospital and labour will be induced” (D)*

*“Your body can not take it anymore … I do not know whether you as a person are the first to die or if it is the baby .... Yes, you could die if the blood pressure does not drop”(D7)*

*“It can be dangerous…. For the baby it might be dangerous. And for mom it’s dangerous for her heart” (D)*

Answers about complications were different from Moroccan participants. One woman was able to identify some of HDP complications. Others were confused between complications of chronic hypertension and HDP.

“*This is a dangerous disease because the woman may die and her child ... the child may be born malformed” (M)*

*“It is dangerous for heart and brain and may cause paralysis” (M)*

#### Sub-theme: Knowledge of treatment for HDP and traditional practices

A wide variety of possibilities were mentioned by the women as possible treatment options. A couple of women knew induction of labour is a way to manage HDP. Also, some women knew that medication is available for treatment. A small number of women mentioned salt restriction, or bed rest or reduce stress for treatment. In Morocco, half of the women thought it was also possible to use alternative medications. In the Netherlands there was one woman.

*“I use lemon and rose water for headaches. I put lemon slices on the forehead” (M)*

*“Garlic is a good treatment for hypertension. My grandmother puts garlic in her ears” (M)*

*“Sometimes headache is caused by the evil eye and fumigation is a good remedy for this” (M)*

*“I think there is no treatment available, perhaps salt restriction?” (D)*

*“Certain food” (D)*

*“There is almost nothing, I believe there can’t be anything. In your pregnancy you would rather not use medication, all you get is paracetamol” (D)*

*“It is important to keep moving, because the first pregnancy I also swelled. Maybe it was because of HDP or because of hormones. So keep moving, and as the dietician said: limited intake of salt, sometimes it’s helpful, but not for everyone. And bed rest, I think. That’s the only thing I can think of now” (D)*

*“I think maybe medication or bed rest” (D)*

*“I think there are things you can eat.. I do not know exactly .. I think that in Morocco, I think there are all kinds of remedies, some herbs. But whether it’s helpful? Some herbal remedies may be.” (D)*

*“Rest and in some cases medication? Stress reduction” (D)*

### Theme 2: Behavior in case of HDP

#### Sub-theme: Practices in case of HDP

When asked about behavior in case of HDP, women were divided between going to health centers and using traditional remedies in Morocco.

*“I must certainly see a health care professional” (M)*

In the Netherlands almost all women would immediately phone the midwife or the general practitioner. Some women thought they would wait for a while, to see if the complaints would disappear spontaneously. All women said that they would contact a healthcare professional.

*“I have no idea. I think, I would call my midwife. But I don’t know for sure? If I doubt about something I always can call the midwife” (D)*

*“I don’t know. I think I would call the hospital” (D)*

*“If I notice something is not okay, than I wait till it is better. But if it persists, then I would call.” (D)*

*“I would immediately visit the doctor” (D)*

*“Immediately call the midwife, and otherwise. Yes, I call her for everything, also if I doubt” (D)*

### Theme 3: Information about HDP

#### Sub-theme: Source of information

The few women who had some knowledge of HDP stated they received the information from a friend, their midwife, or through radio.

*“My friend had this disease, that is why I know lot of things about it. I also received information by the midwife in the health center” (M)*

*“We can learn a lot by listening to the radio” (M)*

But also a few women could not recall any information about HDP at all.

*“No actually not …”(D)*

*“No, no. She [the midwife] has told me nothing about it. I did not receive any information on high blood pressure. I think it’s important; in particular, that it can occur… I like to know…. ”(D)*

In case of complaints in pregnancy most women stated that they would ask their maternity care provider or general practitioner for advice. However, some women would search the internet in order to find useful information.

#### Sub-theme: Content of information

The content of information about HDP received by the few women focused on symptoms and complications of this disease. The women found it important to be well informed. They felt that providing information about headache, abdominal pain, nausea, oedema was an important task for the midwife.

*“Now I know very well symptoms of this disease and the dangerous complications” (M)*

*“The most important thing that I remember is that I must immediately go to the health center for consulting” (M).*

*“The midwife has told me something about when I should call her, but it was not high blood pressure. I think information on HDP is important, I want to know. It is information that helps me to care for the baby. Therefore, this is good as an educational moment.”(D)*

### Theme 4: Suggestions

#### Sub-theme: Important information to know

All women acknowledged the importance of knowledge of this disease because of the possibly dangerous complications. The interviewees stated that information on symptoms, alarm signs, complications and which healthcare provider is the most appropriate person in case of HDP, is the most important information.

All participants in this study raised the importance of communicating about this disease.

“*It is important that all women have information about symptoms of this disease and how to treat it*.” *(M)*

*“I think everyone must know that it is a very dangerous disease*” *(M)*

*“I want to know what I must do if I have such symptoms or alarm signs”* (M)

“*Information must be communicated about the dangerous use of traditional practices*” *(M)*

“*A particular website, if you know that it is safe. Not on forums or other public sites. You read so many things, that are not trustworthy. A website with good information would be important*” *(D)*

#### Sub-theme: Most appropriate tool for the communication of information

**Fig. 1:**
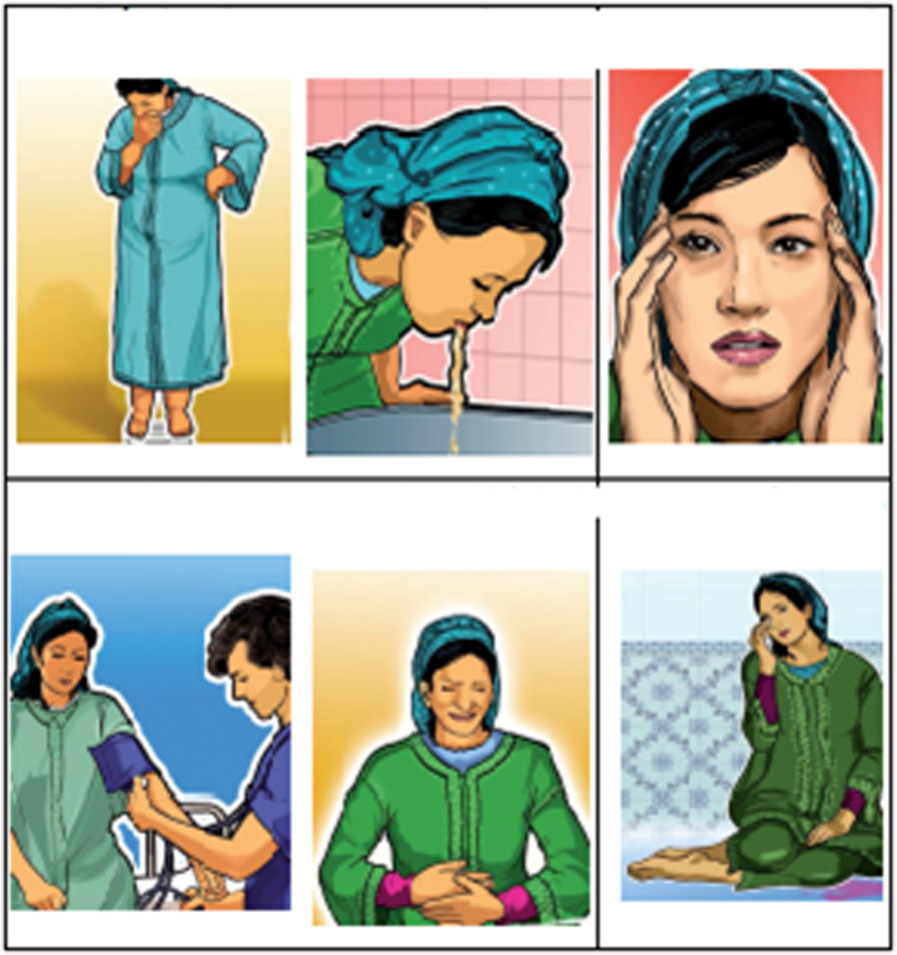
Images of symptoms related to hypertensive disorders in pregnancy, by JICA (Japan International Cooperation Agency). Endnote: These images are not under copyright, they are developed by JICA in cooperation with the Moroccan Ministry of Health to improve maternal health in Morocco. Additional permission for the use of these images is not required

During interviews, we presented to participants a leaflet elaborated by the Ministry of Health in collaboration with Japan International Cooperation Agency (JICA). This pictorial leaflet is used in Moroccan health structures in ‘mother classes’ to educate women about HDP. See Fig. 1.

Women interpreted the images differently. No woman made the connection between reported symptoms and HDP. Women associated the images of nausea with morning sickness in the first trimester of pregnancy. Because there is little knowledge of high blood pressure, women did not associate the image of a vomiting pregnant woman with high blood pressure.

*“I see a picture of a woman who has headaches, vomits and has seizures, but it is unclear whether she is pregnant or not” (M)*

*“Photos show sick women” (M)*

*“I see a pregnant woman with oedema, but it is not indicated what she must do?” (M)*

“*The picture of oedema is not clear. The pregnant belly is not visible on the pictures. The graphic visual complaints are not clear*” *(D)*

“*I think this really is not such a clear picture. It looks like the women is depressed, washed-out, not feeling well.” (D)*

To the question about the most appropriate tool to inform pregnant women about HDP there was almost unanimity on communicating information via movies. Nearly all women recommended a movie or a digital tool. Women in Morocco recommended a movie in Arabic dialect so for women in the rural areas the information would be clear and understandable, widely disseminated and understood. Women in the Netherlands suggested giving attention to this issue during Dutch language lessons.

*“It will be very good if they make movies about this disease” (M)*

*“Information must be in a movie with Moroccan dialect, in order to be understood by illiterate and educated women”(M)*

*“A movie would be ideal” (D)*

## Discussion

In our study, pregnant Moroccan women in Morocco and in the Netherlands were interviewed. Their knowledge of symptoms related to hypertensive disorders of pregnancy was limited or absent. Their fragmented knowledge of hypertension-related symptoms and complications was based on their own experiences or on those of some family members or stories from their social network. Most women stated that they had received none or only little information on symptoms that might be dangerous in pregnancy from their midwives or obstetricians. Several women would look for information on the internet. The internet provides an immediate but frequently unreliable recourse for health and pregnancy related concerns. A single pregnant woman interviewed in Morocco also mentioned having received some information via radio broadcasts. Although some participants had limited knowledge of HDP, they had difficulty reproducing information about symptoms and complications.

The leaflet especially developed to inform pregnant women about danger signs in pregnancy were regarded as unclear.

Health literacy is the degree to which individuals have the capacity to obtain, process, and understand basic health information and services needed to make appropriate health decisions. It comprises a complex set of reading, listening, analytical, decision-making skills, and the ability to apply these skills to health problems [21]. Low health literacy during the perinatal period, and specifically about preeclampsia, may contribute not only to women’s poor understanding of pregnancy and potential fetal health issues, but also to delayed care and poor health outcomes [22, 23]. Indeed, the responses of pregnant women in Morocco about their behavior in case of risk were mixed between seeking help from health professionals or the use of traditional medicines, demonstrating their unfamiliarity with the disease and its impact on their health and that of their fetuses.

Most of the women interviewed in Morocco were illiterate or had a low level of education. Although many studies in Morocco have shown an association between maternal morbidity and education, [24, 25], it appears that the latter is not the most predominant factor in women’s lack of knowledge of HDP. In another context, You et al. [23] argue in their study that women, regardless of literacy level, have a poor understanding of preeclampsia.

Our results with the Moroccan women interviewed in the Netherlands point in the same direction. Although professional guidelines advise informing pregnant women around 23–26 weeks about hypertension-related symptoms [26] none of the interviewed women (whether primigravida or multiparous) could recall these symptoms. It is not clear whether the midwives did not provide the information or that the interviewed women could not recall the information. Our findings are in line with the results of a focus group session performed by the Dutch Organisation of Obstetricians and Gynecologists (NVOG) among women who were referred for hypertension-related problems. Women seemed not to have been informed about symptoms or danger signs by their primary care provider [27].

An explanation for this finding might be the inadequacy of channels and tools used by health professionals to convey information about HDP. Moreover, our results have shown that it is difficult for women to understand the messages conveyed by the HDP brochure currently used in health structures.

Little knowledge of danger signs in pregnancy were also found in a cohort study on severe maternal morbidity. Few of the women had any knowledge of the danger signs during pregnancy. Therefore, they did not interpret their complaints as early signs of complications, especially in cases of pre-eclampsia [21].

Importantly, results from studies by You et al. [28] and Ogunyemi et al. [29] found that these knowledge gaps appear to be modifiable because women who had received information in an appropriate way demonstrated a greater knowledge of eclampsia and preeclampsia.

Jordaan points out that in order to improve maternal health literacy, it is necessary to adapt information to the needs of women and their families in order to empower them to make informed decisions about their health [30]. This recommendation is in line with the suggestions of the women interviewed in our study who expressed a desire to be better informed about the symptoms and warning signs of HDP, as well as the structure and the persons to contact (in case of emergencies). Studies have shown that information tailored to the culture of patients and their level of education contributes to improving women’s understanding of HDP [28, 31]. Strategies can be used by health professionals to increase the knowledge of women with low health literacy such as the use of simple words, replication by women of the information provided, use of audio or video messages and adapted printed materials [19]. In their randomized controlled trial, You et al. compared the contribution of a graphic-based tool that they developed with the tool that was used in health structures by professionals in improving women’s knowledge of preeclampsia [28]. The results were an increase of women’s knowledge. The authors have attributed the success of the graphical tool to its participatory development process that involved both the experts and the women who would use the tool, thus increasing its adaptability and acceptability by the target population.

The women in our study unanimously preferred to communicate information via short movies in the dialectal Arabic language to make it accessible to all categories of women. The movies can be distributed and shared via social media (Facebook, YouTube etc.)

We previously emphasized the importance of education in improving women’s knowledge of HDP. However, effective prevention should go further by including the adoption of disease management behaviors and the search for skilled care at the appropriate time. Midwives can play a part in this strategy, since one of the strengths that midwives possess is educating women regarding health-related matters [22].

The main limitation of this study is that in the Netherlands only low risk women who received midwife led care were interviewed. Although the group of women was diverse, the study might have been strengthened by inclusion of more women under care in different health care settings and with different obstetric histories. For the women in Morocco, the results might not be generalizable to women in other regions and in the private sector because the study took place in one region and in the public sector. Another limitation of the study is that most participants in the Netherlands were highly educated, whereas in Morocco, the majority of the participants were illiterate.

## Conclusions

This study aids understanding about the knowledge of Moroccan pregnant women of hypertension-related symptoms, both in the Netherlands and in Morocco. The lack of knowledge of symptoms related to hypertensive disorders in pregnancy could possibly endanger women’s own health or the health of their babies. To address problems with information about these symptoms, high-quality information is needed that is accessible to women.

## Additional file

Additional File 1: Interview guide for qualitative research into knowledge of hypertensive disorders in pregnancy of Moroccan women in Morocco and in the Netherlands. (DOCX 19 kb)

## Abbreviations

AMSF: Association Marocaine des Sages Femmes
D: Pregnant Moroccan woman living in the Netherlands participating in our study
HDP: Hypertensive disorders in pregnancy
ICM: International Confederation of Midwives
JICA: Japan International Cooperation Agency
KNOV: Dutch Organisation of Midwives
M: Pregnant Moroccan woman living in Morocco participating in our study
MoFA: Ministry of Foreign Affairs, The Netherlands
