# Supplementary material for: Knowledge of hypertensive disorders in pregnancy of Moroccan women in Morocco and in the Netherlands: a qualitative interview study
Source: BMC Pregnancy Childbirth. 2018 Aug 22;18:344. doi: 10.1186/s12884-018-1980-1 (PMC6106822; doi:10.1186/s12884-018-1980-1)
Supplement: Supplementary file 1 — Interview guide for qualitative research into knowledge of hypertensive disorders in pregnancy of Moroccan women in Morocco and in the Netherlands. (DOCX 19 kb) [file 12884_2018_1980_MOESM1_ESM.docx]

**Knowledge of hypertensive disorders in pregnancy of Moroccan women in Morocco and in the Netherlands**

**Interview Guide**

**Introduction:** Self introduction, name and general affiliation

**Length:** max 45-60 min

**Purpose of Interview**

Introduction of the aim of the study and the aim of the interview:

Hypertensive disorders in pregnancy is a common problem in pregnancy. We are interested in what you know about this topic, knowing your views about this problem, with a focus on your experience, and what you know, think or feel about the topics covered.

Ask participants to be open and honest; there are no right or wrong answers. Inform the participants about recording the interview and ask permission their permission.

**Verbal consent:**

Would you like to participate in this interview?

Verbal consent was obtained from the study participant

Verbal consent was NOT obtained from the study participant

**Written consent:**

Ask the study participant to give written consent.

**Interview begins**

Background information

Invite the interviewee to briefly tell me about herself. General information about background:

Age

Residence/area/live with family?

Education

Payed job?

Gravidity

Parity

How many weeks pregnant?

How and in what time reach the hospital?

Experiencing any complications in current pregnancy?

Knowledge

What do you know about high blood pressure in pregnancy?

Who can have it?

Could it be dangerous? For you? For your baby?

When do you think problems with high blood pressure can occur in pregnancy? (whole pregnancy, only at the end?)

What do you know about signs? Symptoms and complications?

What would you do if you had high blood pressure in your pregnancy?

What signs and symptoms for which you need to go to hospital?

What do you know about traditional medication in case of high blood pressure?

Ask in depth about any other signs and symptom for which she needs to phone/contact the midwife/doctor?

Origin of knowledge

How come you know all this?

Who gave you the information?

Did you read about it?

Did you mother, sister or mother in law told you this?

Did your mother or sister or mother in law for example gave you tips and trics in the pregnancy. Did she tell you about what to do use etc in pregnancy and what not to do? Could she explain why?

Most midwives inform their clients. How about your midwife? Did she gave you information?

What did she tell you?

Treatment

Do you know what kind of medicine or treatment you can use in pregnancy for complaints? What would you do?

Prevention

Do you think you can prevent it? How?

Or that it will not happen to you? Why?

In your opinion what do all pregnant women need to know about high blood pressure?

What do all pregnant women need to know?

What would you tell your friend/neighbor?

What would you consider the best way to inform women, to reach all (Moroccan) women?

**Show the participant JICA leaflet on hypertensive disorders in pregnancy**

What do you think about these pictures?

Do you understand the pictures/can you explain what you see?

Do you think it will help in recognizing the symptoms

Do you think it will make her/other women afraid/anxious?

Do you want anything to add?

Do you think this is a good way to inform women.

What, in your opinion, would be the best way to inform women?
